# Enhanced fear limits behavioral flexibility in *Shank2*-deficient mice

**DOI:** 10.1186/s13229-022-00518-1

**Published:** 2022-10-03

**Authors:** Miru Yun, Eunjoon Kim, Min Whan Jung

**Affiliations:** 1 Department of Biological Sciences, Korea Advanced Institute of Science and Technology, Daejeon, 34141 Korea; 2 Center for Synaptic Brain Dysfunctions, Institute for Basic Science, Daejeon, 34141 Korea

**Keywords:** Shank2, Reversal learning, Fear, Classical conditioning

## Abstract

**Background:**

A core symptom of autism spectrum disorder (ASD) is repetitive and restrictive patterns of behavior. Cognitive inflexibility has been proposed as a potential basis for these symptoms of ASD. More generally, behavioral inflexibility has been proposed to underlie repetitive and restrictive behavior in ASD. Here, we investigated whether and how behavioral flexibility is compromised in a widely used animal model of ASD.

**Methods:**

We compared the behavioral performance of *Shank2*-knockout mice and wild-type littermates in reversal learning employing a probabilistic classical trace conditioning paradigm. A conditioned stimulus (odor) was paired with an unconditioned appetitive (water, 6 µl) or aversive (air puff) stimulus in a probabilistic manner. We also compared air puff-induced eye closure responses of *Shank2*-knockout and wild-type mice.

**Results:**

Male, but not female, *Shank2*-knockout mice showed impaired reversal learning when the expected outcomes consisted of a water reward and a strong air puff. Moreover, male, but not female, *Shank2*-knockout mice showed stronger anticipatory eye closure responses to the air puff compared to wild-type littermates, raising the possibility that the impairment might reflect enhanced fear. In support of this contention, male *Shank2-*knockout mice showed intact reversal learning when the strong air puff was replaced with a mild air puff and when the expected outcomes consisted of only rewards.

**Limitations:**

We examined behavioral flexibility in one behavioral task (reversal learning in a probabilistic classical trace conditioning paradigm) using one ASD mouse model (*Shank2*-knockout mice). Thus, future work is needed to clarify the extent to which our findings (that enhanced fear limits behavioral flexibility in ASD) can explain the behavioral inflexibility associated with ASD. Also, we examined only the relationship between fear and behavioral flexibility, leaving open the question of whether abnormalities in processes other than fear contribute to behavioral inflexibility in ASD. Finally, the neurobiological mechanisms linking *Shank2*-knockout and enhanced fear remain to be elucidated.

**Conclusions:**

Our results indicate that enhanced fear suppresses reversal learning in the presence of an intact capability to learn cue-outcome contingency changes in *Shank2*-knockout mice. Our findings suggest that behavioral flexibility might be seriously limited by abnormal emotional responses in ASD.

**Supplementary Information:**

The online version contains supplementary material available at 10.1186/s13229-022-00518-1.

## Background

Autism spectrum disorders (ASD) are developmental disorders that are associated with a diverse array of symptoms, including impaired social interaction and communication as well as repetitive and restrictive patterns of behavior [1]. Many genes implicated in ASD are expressed broadly in the cerebral cortex, and their mutations may lead to aberrant circuit development in widespread cortical areas [2–4]. Given that the cerebral cortex (especially frontal cortical areas such as the orbitofrontal, anterior cingulate, and dorsolateral prefrontal cortices) plays a key role in the adaptive control of behavior [5–10], mutations in genes that play important roles in cortical development may lead to compromised behavioral flexibility in adults. People with ASD often show impairments in tasks requiring cognitive flexibility, such as the Wisconsin card sorting test [11, 12], and impaired cognitive flexibility has been proposed to underlie repetitive and restrictive patterns of behavior in this disorder [13]. People with ASD also show impairments in reversal learning, and their performance in reversal learning correlates with clinical ratings of restricted and repetitive behavior [14] and everyday symptoms of behavioral inflexibility [15]. Impaired behavioral flexibility has also been reported in mouse models of ASD [16–30]. Thus, a growing body of studies indicates that impaired behavioral flexibility is present and correlated with repetitive and restrictive behaviors in ASD, raising the possibility that repetitive and restrictive patterns of behavior associated with ASD might be manifestations of impaired flexibility.

Shank2 (SH3 and multiple ankyrin repeat domains 2), which is a multi-domain scaffolding protein enriched in the postsynaptic density of excitatory synapses [31, 32], is strongly implicated in ASD [33]. Genetic variations of the *SHANK2* gene have been identified in ASD [34–58]. Moreover, mutations/deletions in the *Shank2* gene lead to a diverse array of behavioral changes in mice; some of these mutant mice show impaired social interaction and repetitive behavior, which are core symptoms of ASD [59–71]. Mice with *Shank2* gene alterations have been used widely to investigate neurobiological mechanisms of ASD, and substantial amounts of behavioral and neural data have been accumulated. However, the relationship between *Shank2* mutations and behavioral flexibility is largely unknown.

In the present study, we investigated whether and how behavioral flexibility is compromised in *Shank2-*KO mice lacking exons 6 and 7 [60] by subjecting them to reversal learning under a probabilistic classical conditioning paradigm. In this task, two different odor cues were paired with a reward (water) or a punishment (air puff) probabilistically over a 1-s delay (trace classical conditioning), and anticipatory licking responses during the delay period were measured as an index for learning. Numerous studies have indicated that the orbitofrontal cortex plays a crucial role in reversal learning [72, 73]. We previously showed that an intact medial prefrontal cortex is also involved in probabilistic reversal learning in mice [74]. Shank2 is expressed broadly in the brain including the orbitofrontal and medial prefrontal cortices [75]. We have also shown various physiological abnormalities in the medial prefrontal cortex of *Shank2*-KO mice [67, 68]. These findings suggest a potential link between *Shank2*-KO and a reversal learning deficit.

We examined reversal learning of both male and female *Shank2*-KO mice because people with ASD are four times more frequent in males than females [76] and male–female differences in various behavioral, synaptic, molecular, and neuroanatomical phenotypes have been detected in mouse models of ASD [77–82]. We also examined reversal learning in juvenile *Shank2*-KO mice because ASD is characterized by early manifestations of symptoms and mouse models of ASD, including *Shank2*-KO mice, frequently show differential phenotypes at different postnatal stages [60, 67, 83]. We found that male *Shank2*-KO mice, both adult and juvenile, but not female adult *Shank2*-KO mice, show impaired reversal learning only when a strong air puff was used as an unconditioned stimulus (US). We also found that male, but not female, *Shank2-*KO mice display abnormally heightened fear responses to the strong air puff. The results suggest that abnormal emotional responses may limit behavioral flexibility in ASD under certain circumstances.

## Methods

### Subjects

We used *Shank2-*KO mice that harbor deletions in exons 6 and 7 of *Shank2* and thus mimic the ASD-related microdeletion of exons 6 and 7 in human *SHANK2* [60]. We used 45 adult (12–24 weeks old) male *Shank2-*KO mice, 45 adult male wild-type (WT) littermates, 17 adult female *Shank2*-KO mice, 12 adult female WT littermates, 22 juvenile (P30–45) male *Shank2*-KO mice, and 20 juvenile male WT littermates in this study. Different groups of mice were tested in three different versions of reversal learning (Tasks 1–3; see below). Some of the mice were tested for air puff-induced eye closure responses before being tested in reversal learning (Additional file 1: Table S1). The mice had a C57BL/6 N background and were characterized by PCR genotyping as previously reported [60, 67]. For classical conditioning, the mice were water-deprived. They were allowed to drink water only during the task (total amount per session, Tasks 1 and 2, ~ 0.9 ml; Task 3, ~ 1.2 ml) unless their body weights fell below 80% of their initial body weights. Additional water (1–4 ml) was provided 1 h after the task to those mice whose body weights fell below 80% of their initial body weights when assessed immediately after task completion. Mice used to measure eye closure responses to air puff were fed ad libitum. The estrous cycle of female mice was not assessed. All mice were housed individually and all experiments were performed during the dark phase of a 12 h light/dark cycle.

### Surgery

General anesthesia was induced in mice with isoflurane (3.0% in 100% [v/v] oxygen) inhalation for 5 min. The anesthetized mice were head-fixed on a stereotaxic system, their head skin was removed to expose the skull, and the concentration of isoflurane was lowered to 1.5–2.0%. A customized aluminum head plate was briefly sterilized with 70% ethanol and placed on the skull near the lambdoid suture [74, 84]. The head plate was fixed by sterilized screws (M1 × 3 mm) and covered with dental cement. The mice were allowed to recover > 1 week before behavioral training began.

### Probabilistic trace classical conditioning

**Fig. 1:**
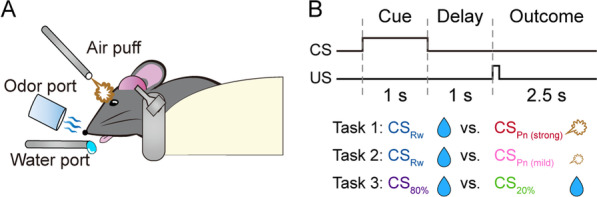
Behavioral task. **A** The experimental setting. Head-fixed mice performed a probabilistic classical conditioning task. **B** Task schematic. A conditioned stimulus (CS; odor) was delivered for 1 s, there was a 1-s delay, and then an appetitive (water, 6 µl) or aversive (air puff) US was delivered in a probabilistic manner. Each CS was paired with 75% water (CS_Rw_) or 75% strong air puff (CS_Pn(strong)_) in Task 1, with 75% water or 75% mild air puff (CS_Pn(mild)_) in Task 2, and with 80% water (CS_80%_) or 20% water (CS_20%_) in Task 3

All mice were trained in a probabilistic trace classical conditioning task under head fixation (Fig. 1A) as previously described [74]. The animal’s head was fixed to a custom-built metal holder. A water port (a blunt 17-gauge needle) was placed slightly below the animal’s nose, an air puff port (a blunt 18-gauge needle) was placed 3–5 mm away from the animal’s left eye, and an odor port (silicon tube; diameter, 8 mm) was placed slightly above the animal’s nose. Four different odors (citrus, isoamyl acetate, L-carvone, and 1-butanol) were dissolved in mineral oil (1:1000, v:v) and delivered to the animal using an air circulation system. The animal’s licking behavior was detected by an infrared light sensor placed adjacent to the water port.

The behavioral phases consisted of habituation, acquisition, and reversal. For each animal, two odors were selected randomly from the four possible odors and used in the acquisition and reversal phases. For habituation, a small amount of water (6 µl) was provided from the water port initially every 5 s without an odor cue (~ 50 trials, day 1). Then the same amount of water was provided 1 s after the delivery of an odor cue (1 s) that was different from those used in the acquisition and reversal phases, and a 2.5–4.5 s inter-trial interval (uniform random distribution) was imposed. The habituation phase lasted 1–3 sessions (~ 400 trials per session).

In the acquisition phase, an odor cue randomly chosen from two different odors was delivered for 1 s, there was a delay of 1 s, and an associated outcome was delivered with a given probability (Fig. 1B). An inter-trial interval (2.5–4.5 s, uniform random distribution) was then imposed before the next trial began. The mice were trained for three daily sessions of 400 trials in the acquisition phase. We used three different sets of cue-outcome contingencies. A reward-predicting conditioned stimulus (CS_Rw_) and a punishment-predicting conditioned stimulus (CS_Pn_) were paired with water (6 µl) and air puff, respectively, as follows:

- Task 1 (strong air puff): CS_Rw_ (75% delivery of water) and CS_Pn_ (75% delivery of strong air puff; 100 ms, 3 psi).
- Task 2 (mild air puff): CS_Rw_ (75% delivery of water) and CS_Pn_ (75% delivery of mild air puff; 5 ms, 3 psi).
- Task 3 (no air puff): CS_80%_ (80% delivery of water) and CS_20%_ (20% delivery of water).

In the reversal phase, the mice were first trained with the same cue-outcome contingency as in the acquisition phase until they reached the criterion for reversal onset, which was when the smoothed anticipatory lick rate (moving average of 20 trials) was significantly different between two cues (CS_Rw_ versus CS_Pn_ or CS_80%_ versus CS_20%_) in ≥ 75 of the prior 100 analysis windows. Then the cue-outcome contingency of the acquisition phase was reversed. For Tasks 1 and 2, the reward-predicting cue before reversal was paired with punishment after reversal (CS_Rw→Pn_) and the punishment-predicting cue before reversal was paired with a reward after reversal (CS_Pn﻿→Rw_). For Task 3, the cue predicting 80% reward delivery before reversal was paired with 20% reward delivery after reversal (CS_80%﻿→20%_) and the cue predicting 20% reward delivery before reversal was paired with 80% reward delivery after reversal (CS_20%﻿→80%_). Each mouse was trained until it met the reversal criterion, which took 1–5 daily sessions (400 trials per session). The reversal criterion was five consecutive trials after cue-outcome contingency reversal during which the smoothed anticipatory lick rate (moving average of 25 trials) was significantly higher (*t* test, *p* < 0.05) following CS_Pn﻿→Rw_ than CS_Rw﻿→Pn_ (or CS_20%﻿→80%_ than CS_80%﻿→20%_).

Performance in reversal learning was assessed in two ways. The first measure was the number of trials needed to reach the reversal criterion. Note that this measure was obtained over a variable number of sessions (1 to 5) across animals. The second measure was the relative cue-dependent lick frequency in the first reversal session. Given that the number of trials after cue-outcome contingency reversal in the first session varied between 50 and 289 across all animals and all tasks (Task 1, 62–270; Task 2, 50–283; Task 3, 64–289), we equalized the number of first-session trials to the smallest number for a given task (Task 1, 62; Task 2, 50; Task 3, 64) and then estimated the lick difference index (LDI) using the last 10 equalized trials. The LDI was calculated as follows:

$$LDI \, = \, \left( {A \, {-} \, B} \right)/\left( {A + B} \right),$$

where *A* is the anticipatory lick rate during the delay period in initially more rewarding trials (CS_Rw﻿→Pn_ in Tasks 1 and 2 and CS_80%﻿→20%_ in Task 3) and *B* is the anticipatory lick rate during the delay period in initially less rewarding trials (CS_Pn﻿→Rw_ in Tasks 1 and 2 and CS_20%﻿→80%_ in Task 3).

### Eye closure response

**Fig. 2:**
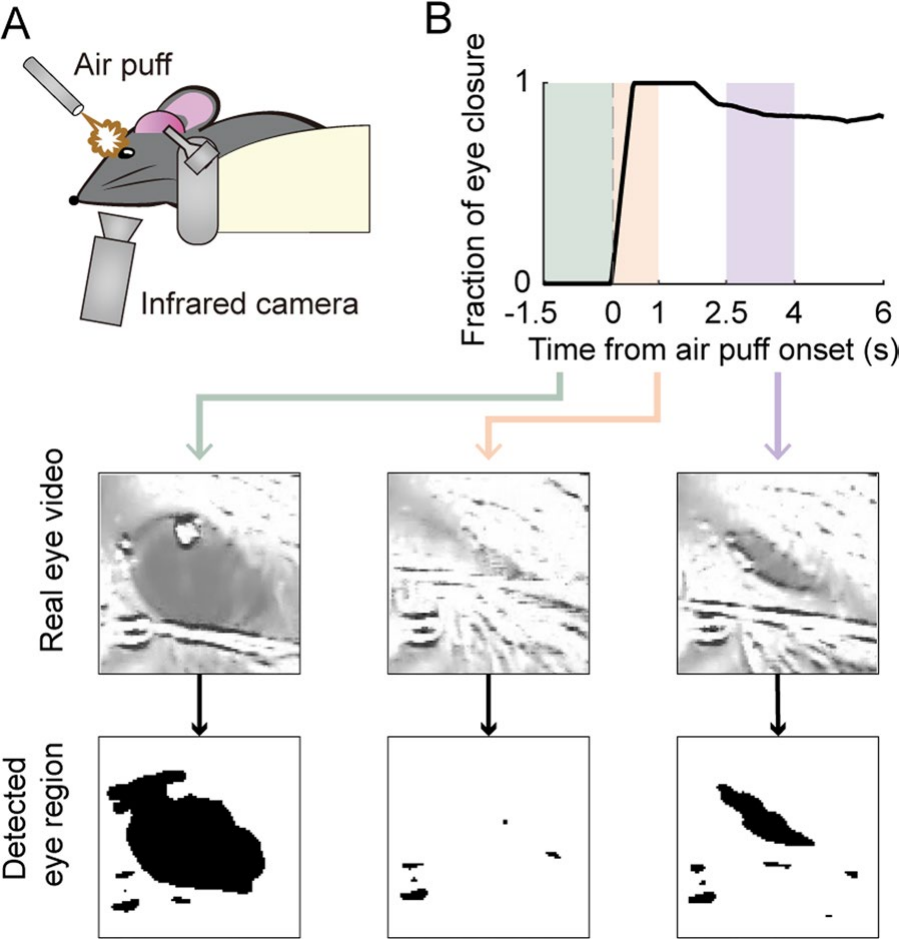
Measurement of eye closure. The eye closure response to air puff was estimated by measuring pupil diameter with an infrared camera. **A** Schematic for the experimental setting. **B** A sample eye closure response to an air puff. Video images and detected eye regions before and after air puff delivery are shown at the bottom

The mice were habituated to the head-fixed experimental setting for 1 h per day for 3 days. Adult male mice received different durations (5, 10, 50, 100 ms) and intensities (3, 7, 15, 30 psi) of air puff (total, 16 combinations) five times each (Fig. 2A). Adult female mice were tested with three combinations of air puff duration and intensity (100 ms at 3 psi; 5 ms at 3 psi; and 100 ms at 30 psi). Consecutive air puffs were separated by 9–11-s intervals (uniform random distribution). The animal’s eye closure response was quantified by measuring the area of the left pupil with an infrared camera (IDIS Co., Ltd., South Korea) at 30 Hz, as previously described [74, 85, 86]. Briefly, raw video images were converted to grayscale images, Gaussian-filtered (*σ* = 2 pixels), and thresholded to generate binary masked images. The pupil area was measured from the binary masked images and then normalized between 0 (fully open eye) and 1 (fully closed eye; Fig. 2B).

### Pharmacological rescue

D-cycloserine (DCS; Sigma, C6880) was dissolved in filtered saline (6 mg/ml). DCS (20 mg/kg) or the same volume of saline was injected to the animal intraperitoneally at 30 min before measurement of eye closure responses. All mice were tested twice, once following DCS injection and once following saline injection. The sequence of drug injection was counterbalanced across mice. We tested three combinations of air puff duration and intensity (100 ms at 3 psi; 5 ms at 3 psi; and 100 ms at 30 psi), which enabled us to complete the test within the half-life of DCS (23 min).

### Statistical analysis

All statistical tests were performed with MATLAB (version R2017a) and SPSS (version 25.0). Group comparisons were performed with Student’s *t* test and multiple-way ANOVA (independent and mixed) with Bonferroni post hoc tests. All statistical tests were two-tailed. Statistical significance was accepted at *p* < 0.05. All data are expressed as mean ± SEM.

## Results

### Impaired reversal learning in male Shank2-KO mice

**Fig. 3:**
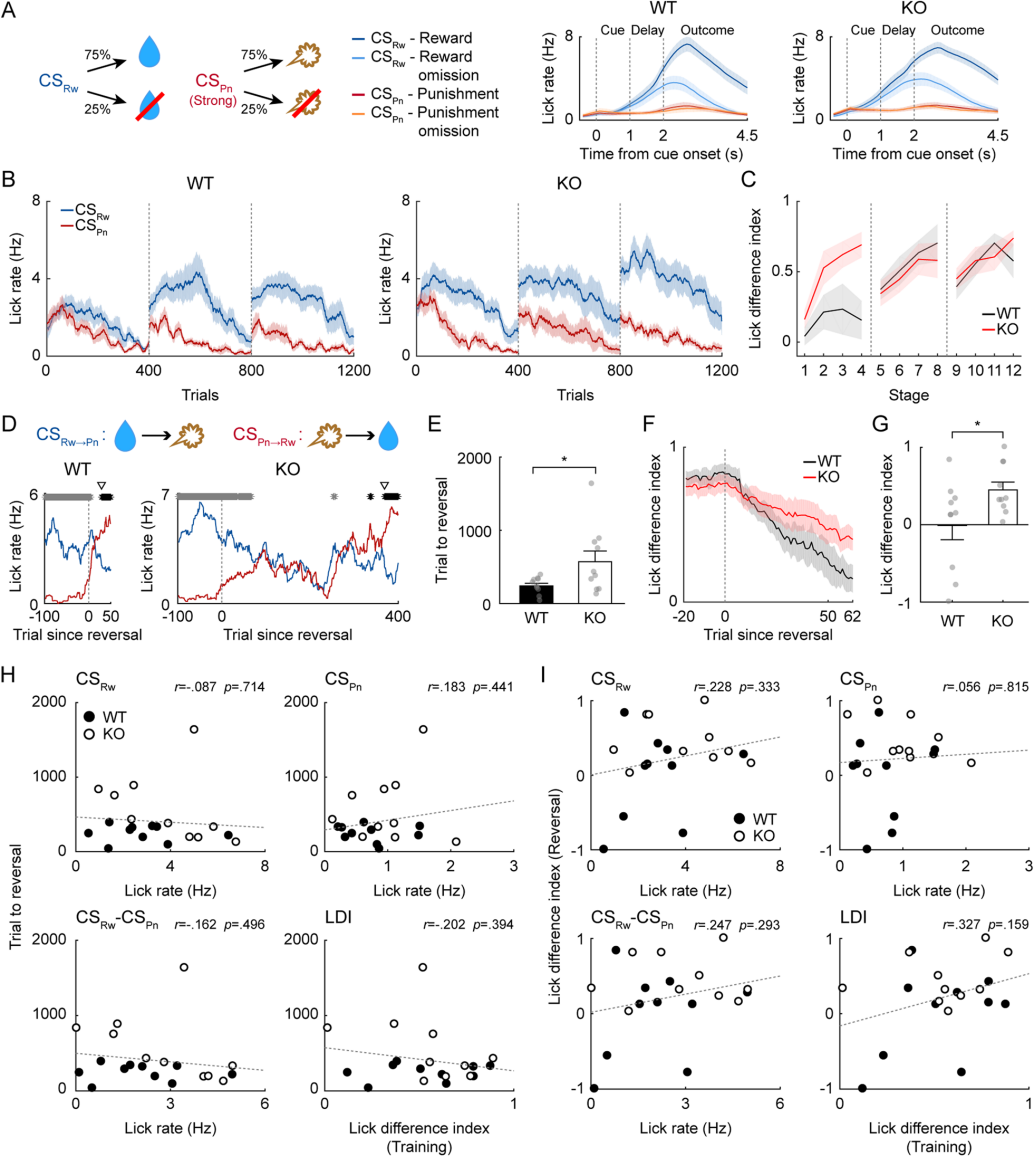
Reversal learning is impaired in adult male *Shank2-*KO mice. **A** Left, two odor cues, CS_Rw_ and CS_Pn_, were paired with 75% reward (water, 6 µl; blue) and 75% strong air puff (100 ms, 3 psi; red), respectively (Task 1). Right, mean licking responses (lick density functions, *σ* = 100 ms) of WT (left; *n* = 10) and *Shank2-*KO (right; *n* = 10) mice during the last acquisition session. Trials were grouped according to CS and outcome. **B** Mean delay period anticipatory lick rates in response to CS_Rw_ (blue) or CS_Pn_ (red) during initial acquisition (three sessions). **C** The LDI of WT (black) and *Shank2*-KO (red) mice in each stage (100 trials) during initial acquisition. **D** Sample reversal learning sessions. Blue and red lines indicate anticipatory licking responses to CS_Rw→Pn_ and CS_Pn→Rw_ cues, respectively, in a moving average of 25 trials. Gray and black asterisks at the top indicate significantly higher anticipatory licking response to the CS_Rw→Pn_ (gray) or CS_Pn→Rw_ (black) cue compared to the other cue, in a moving window of 25 trials (*p* < 0.05, *t* test). Open triangles indicate the first trial since meeting the cue-outcome contingency reversal criterion. **E** The number of trials required to exceed the reversal criterion. **F** The LDI during the first reversal session (moving average of 25 trials). **G** The LDI during the last 10 trials in **F**. **H, I** Relationships between reversal learning performance (ordinate; **H**, the number of trials needed to reach the reversal criterion; **I**, LDI during the first reversal session [last 10 trials in F]) and the mean anticipatory lick rate in CS_Rw_ trials, that in CS_Pn_ trials, their difference, and the LDI during the last acquisition session (abscissa). Gray dashed lines represent least-squares linear fit. Circles indicate individual animal data **E**, **F**, **H** and **I**. Squares and bar graphs **B**, **C**, **E** and **H** present the mean across 10 mice. Shading and error bars indicate the SEM across 10 mice **A**–**C**, **E**, **G** and **H**. *p < 0.05, *t* test

We first tested adult (12–24-week-old) male mice (10 WT and 10 *Shank2-*KO) on Task 1, in which one odor cue was paired with a small amount of water (6 µl) and another with an air puff (100 ms, 3 psi), each with 75% probability (Fig. 3A). The mice were trained in the task for three daily sessions of 400 trials (acquisition phase). We used the anticipatory lick rate during the delay period (1 s) as an index for discrimination between two odor cues throughout the study. The anticipatory lick rate diverged rapidly (in < 100 trials) according to CS during the first session (Fig. 3B). Overall, the anticipatory lick rate decreased gradually within each session, possibly reflecting a gradual decrease in thirst. Nevertheless, both genotypes showed higher anticipatory lick rates in CS_Rw_ trials than in CS_Pn_ trials throughout the three training sessions (Fig. 3B). We divided each session into four stages (100 trials each) and subjected the LDI (see Section “Methods”) to a two-way mixed ANOVA (Fig. 3C). We found a significant main effect of training (*F*_*(11,198)*_ = 8.014; *p* = 1.7 × 10^–11^) along with a significant training × genotype interaction effect (*F*_*(11,198)*_ = 3.318; *p* = 3.3 × 10^–4^; main effect of genotype, *F*_*(1,198)*_ = 1.193, *p* = 0.289). Post hoc Bonferroni tests revealed that the LDI was significantly higher in KO mice than in WT mice in the fourth stage of the first session (stages 1–4; *p* = 0.211, 0.083, 0.073 and 0.004, respectively; second session, *p* values > 0.2; third session, *p* values > 0.2). These results indicate that although the initial learning rate was faster in *Shank2-*KO mice than in WT mice, both genotypes were over-trained to discriminate between CS_Rw_ and CS_Pn_.

The mice were then trained until they reached the reversal criterion over 1–5 sessions (400 trials per daily session). The dynamic of lick rate changes during reversal varied widely across individual mice. The number of trials required to reach the reversal criterion differed significantly between WT and *Shank2-*KO mice (240.3 ± 35.7 and 570.7 ± 146.6 trials, respectively; *t* test, *t*_*(18)*_ = 2.190, *p* = 0.042; Fig. 3D, E). The mice sometimes consumed water in the subsequent trial rather than during the inter-trial interval following a rewarded trial. To rule out the influence of such invalid anticipatory licks, we deleted the trials during which the mice consumed water delivered in the previous trial (215 out of 8115 trials; 2.65%) and determined the number of trials needed to reach the reversal criterion. This analysis also found that there was a significant difference between WT and *Shank2-*KO mice in the number of trials needed to reach the reversal criterion (240.3 ± 35.7 and 571.2 ± 146.7 trials, respectively; *t* test, *t*_*(18)*_ = 2.190, *p* = 0.042).

To test whether reversal learning was influenced by the level of initial training, we examined the relationship between the number of trials needed to reach the reversal criterion and the number of licks in CS_Rw_ trials, the number of licks in CS_Pn_ trials, the difference in the number of licks between CS_Rw_ and CS_Pn_ trials, and the LDI during the last acquisition session. We found that there was no significant relationship between these measures and the number of trials needed to reach the reversal criterion (Fig. 3H). This indicates that the difference in reversal learning between WT and *Shank2*-KO mice cannot be accounted for by different levels of initial learning.

We also found that the LDI during the first reversal session (calculated using trials #53–62) differed significantly between WT and *Shank2-*KO mice (-0.010 ± 0.184 and 0.449 ± 0.101, respectively; *t* test, *t*_*(18)*_ = 2.189, *p* = 0.042; Fig. 3F, G). No significant relationship was found between the LDI and the number of licks in CS_Rw_ trials, the number of licks in CS_Pn_ trials, the difference in the number of licks between CS_Rw_ and CS_Pn_ trials, or the LDI during the last acquisition session (Fig. 3I). Together, these results indicate that reversal learning is slower in *Shank2-*KO mice than in WT mice.

### Impaired reversal learning in juvenile male Shank2-KO mice

**Fig. 4:**
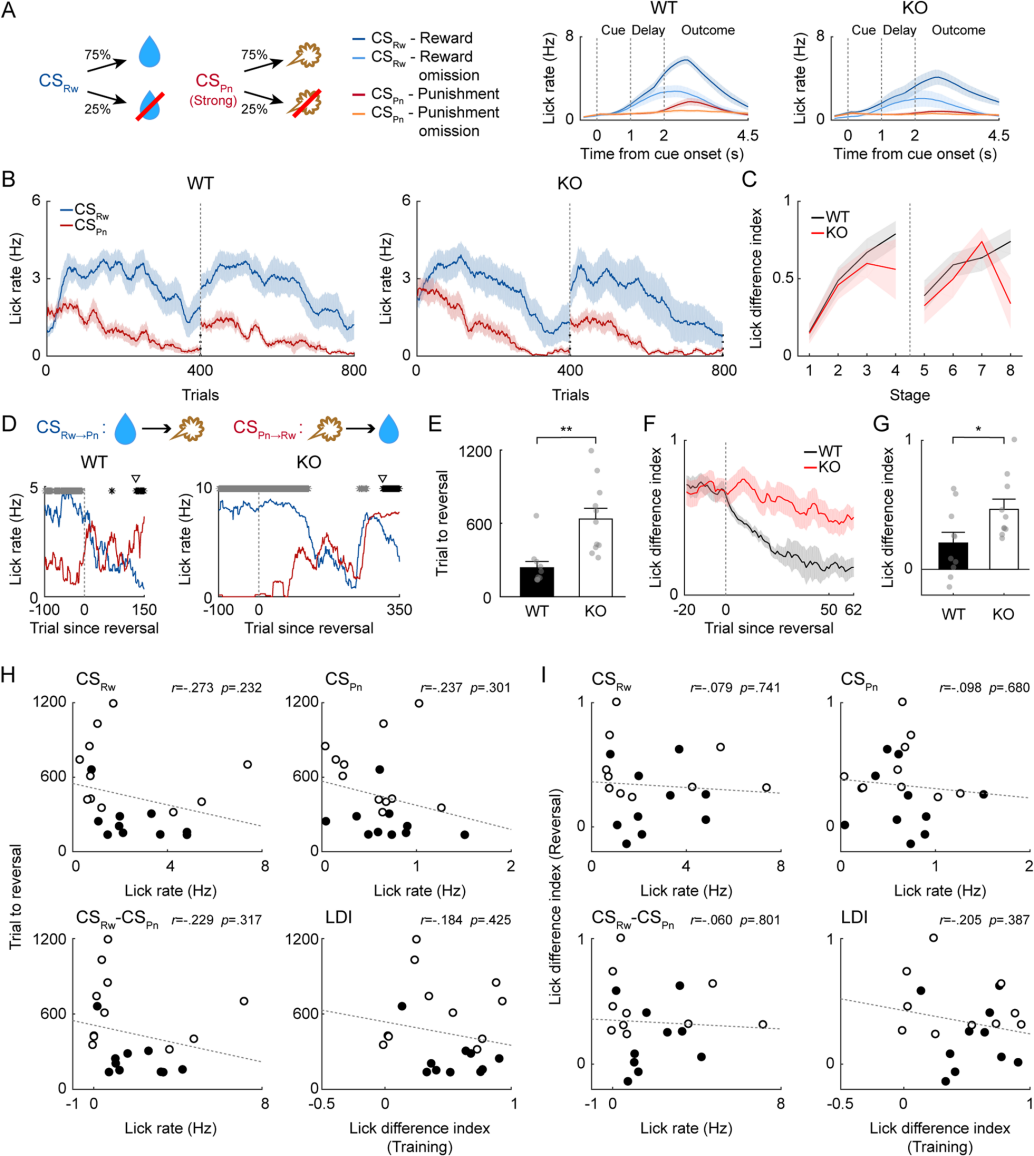
Reversal learning is impaired in juvenile male *Shank2-*KO mice. Results of reversal training with a strong air puff (Task 1) in juvenile male WT (*n* = 10) and *Shank2*-KO (*n* = 11) mice. The results are presented as described for Fig. 3

Given that ASD is a neurodevelopmental disorder and people with ASD may show signs of behavioral inflexibility in childhood [87–92], we tested whether juvenile *Shank2-*KO mice also show deficits in reversal learning. Toward this end, juvenile (P30–45) male mice (10 WT and 10 *Shank2-*KO) were tested in Task 1 as described for adult male mice (Fig. 4A). The mice were trained for three daily sessions during the initial acquisition phase; unfortunately, however, the third-session data were lost due to a procedural error. We therefore assessed initial learning based on the first two sessions. The anticipatory lick rate diverged rapidly according to CS in the first session, and this difference was maintained in the second session (Fig. 4B). Two-way mixed ANOVA of LDI revealed that there was a significant main effect of training (*F*_*(7,126)*_ = 8.400; *p* = 2.1 × 10^–8^), but no significant main effect of genotype (*F*_*(1,126)*_ = 0.601; *p* = 0.448) or training × genotype interaction effect (*F*_*(7,126)*_ = 1.628; *p* = 0.133; Fig. 4C). Although we could not examine the animals’ behavior during the third training session, the mice showed significantly different anticipatory licking responses between CS_Rw_ and CS_Pn_ trials at the outset (first 100 trials) of the first reversal session before reversal onset, and their rates did not differ significantly from those of the corresponding adult male mice (Additional file 1: Fig. S1). Moreover, the number of trials needed to reach the reversal onset criterion did not differ significantly from that of the corresponding adult male mice (Additional file 1: Fig. S1). These results indicate that the juvenile WT and *Shank2*-KO mice were well trained to discriminate between CS_Rw_ and CS_Pn_.

Upon CS-US contingency reversal (2–4 daily sessions of 400 trials until the reversal criterion was reached), the number of trials needed to reach the reversal criterion differed significantly between the juvenile male WT and *Shank2-*KO mice (236.5 ± 50.6 and 634.9 ± 88.2 trials, respectively; *t* test, *t*_*(19)*_ = 2.189, *p* = 0.001; Fig. 4D, E; similar results were obtained after deletion of trials in which previously delivered water was consumed; 306 out of 8945 trials; 3.42%; *t* test, *t*_*(19)*_ = 3.428, *p* = 0.003). The LDI during the first reversal session (calculated using trials #53–62) also differed significantly between the two genotypes (0.203 ± 0.084 and 0.465 ± 0.079, respectively; *t* test, *t*_*(18)*_ = 2.282, *p* = 0.035; Fig. 4F, G). No significant relationship was found between these reversal learning measures (the number of trials needed to reach the reversal criterion and the LDI) and the number of licks in CS_Rw_ trials, the number of licks in CS_Pn_ trials, the difference in the number of licks between CS_Rw_ and CS_Pn_ trials, or the LDI during the last acquisition session (Fig. 4H, I). These results indicate that, as seen for adult males, juvenile male *Shank2-*KO mice were impaired in the reversal learning task studied herein.

### Intact reversal learning in female Shank2-KO mice

**Fig. 5:**
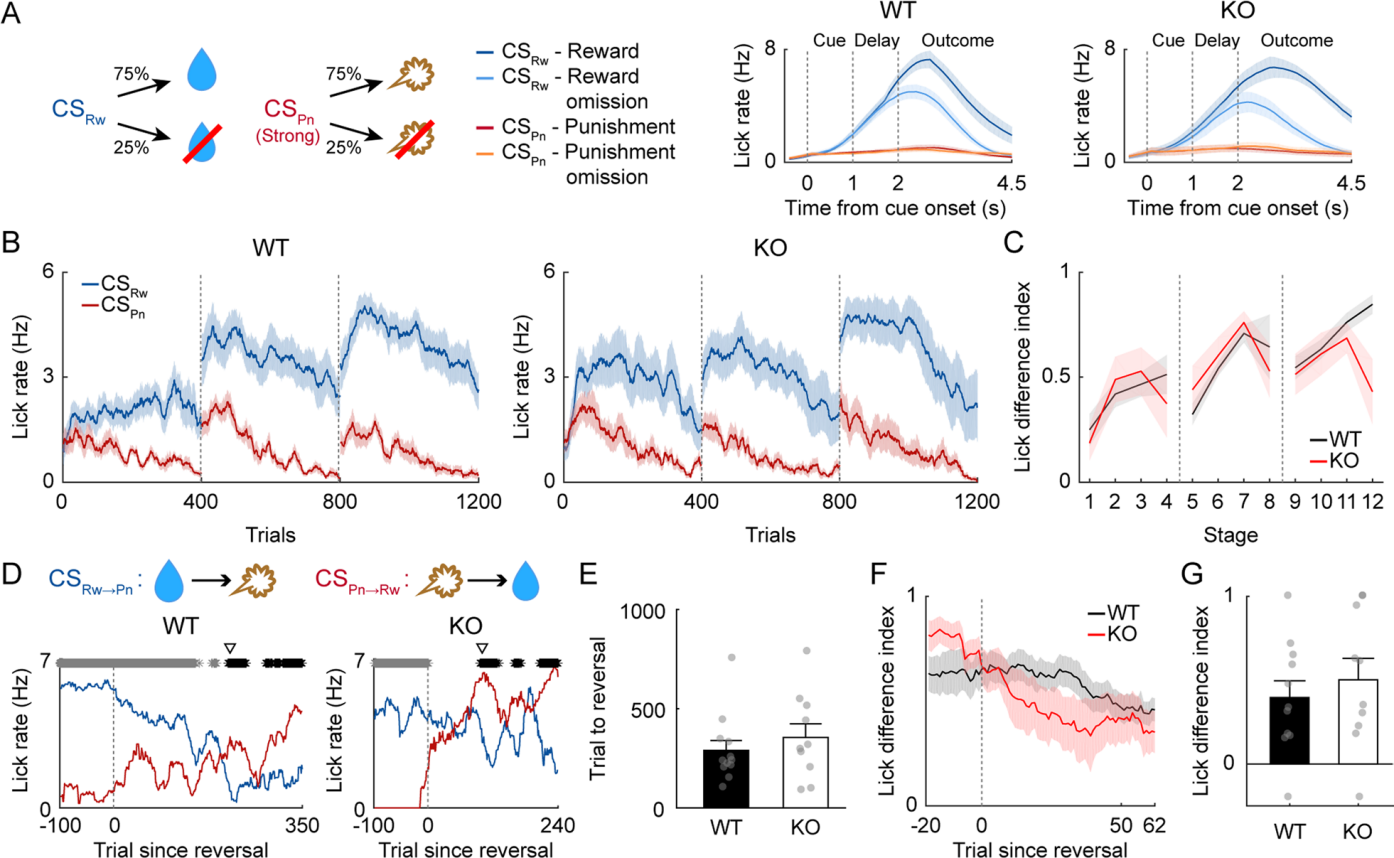
Intact reversal learning in female *Shank2-*KO mice. Results of reversal training with a strong air puff (Task 1) in adult female WT (*n* = 12) and *Shank2*-KO (*n* = 10) mice. The results are presented as described for Fig. 3A–G

Because the prevalence of ASD is strongly male-biased [76], we examined whether adult (12–24 weeks old) female *Shank2-*KO mice also show reversal learning deficits in Task 1 (Fig. 5A). Adult female WT (*n* = 12) and *Shank2-*KO (n = 10) mice showed preferential anticipatory licking in response to CS_Rw_ compared to CS_Pn_ throughout the initial training sessions (Fig. 5B). Two-way mixed ANOVA of LDI revealed that there was a significant main effect of training (*F*_*(11,220)*_ = 6.812; *p* = 7.7 × 10^–10^), but no significant main effect of genotype (*F*_*(1,220)*_ = 0.392; *p* = 0.538) or training × genotype interaction effect (*F*_*(11,220)*_ = 1.611; *p* = 0.097; Fig. 5C).

Upon CS-US contingency reversal (2–4 daily sessions of 400 trials until reaching the reversal criterion), we found that there was no significant difference in the number of trials needed to reach the reversal criterion between female WT and *Shank2-*KO mice (387.9 ± 45.0 and 386.9 ± 77.2 trials, respectively; *t* test, *t*_*(20)*_ = 0.012, *p* = 0.991; Fig. 5D, E; similar results were obtained after deletion of trials in which previously delivered water was consumed; 347 out of 8598 trials; 4.04%; *t* test, *t*_*(20)*_ = 0.408, *p* = 0.687). We also failed to find a significant difference in the LDI (calculated using trials #53–62 of the first reversal session; 0.396 ± 0.100 and 0.502 ± 0.127, respectively; *t* test, *t*_*(19)*_ = 0.661, *p* = 0.516; Fig. 5F, G). These reversal learning measures showed no significant relationship with the number of licks in CS_Rw_ trials, the number of licks in CS_Pn_ trials, the difference in the number of licks between CS_Rw_ and CS_Pn_ trials, or the LDI during the last acquisition session (Additional file 1: Fig. S2). These results indicate that the reversal learning of female *Shank2-*KO mice is intact.

### Enhanced eye closure responses in male Shank2-KO mice

**Fig. 6:**
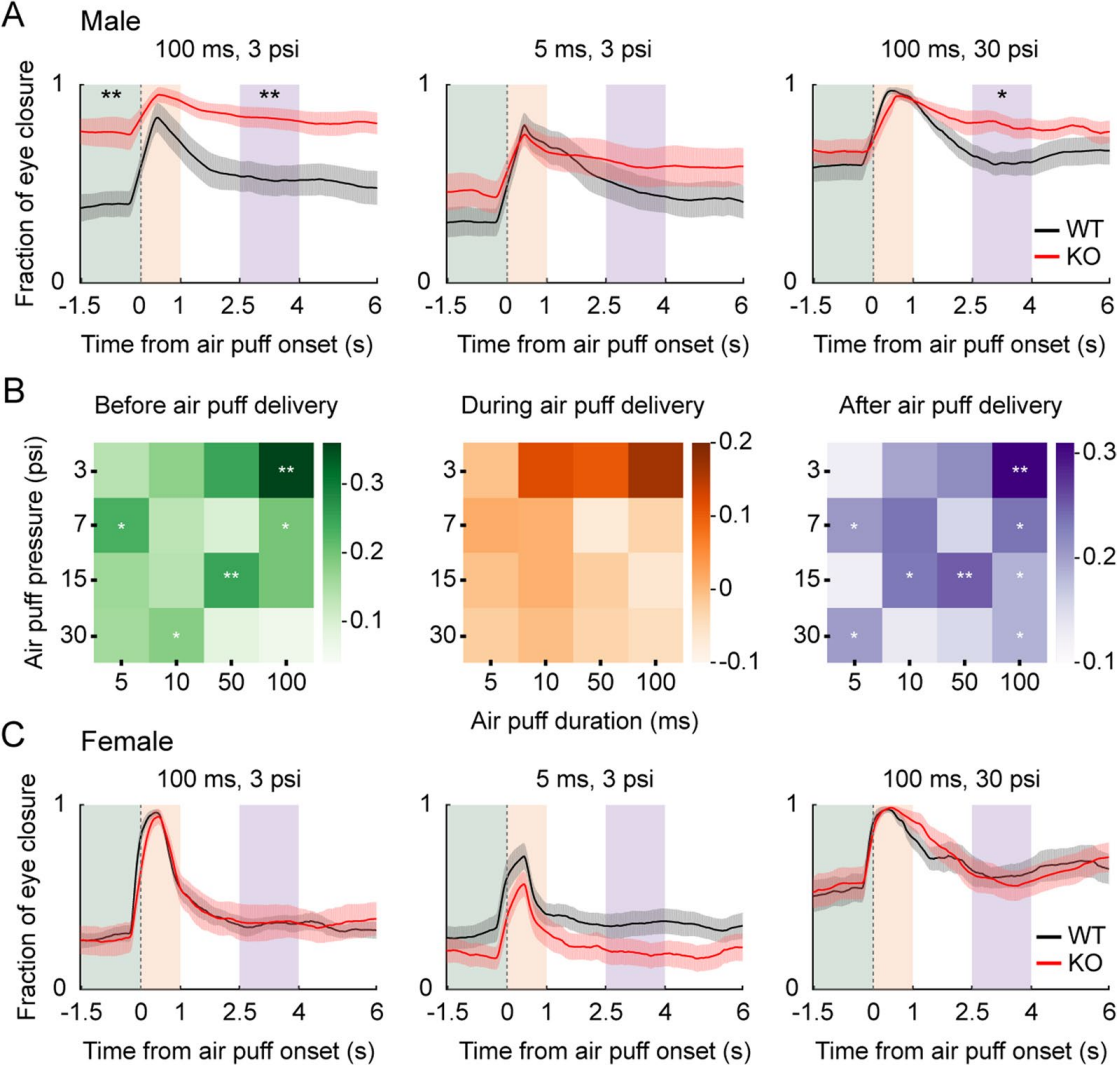
Eye closure response to air puff differs between male, but not female, WT and *Shank2-*KO mice. **A** Eye closure responses (average of 5 trials) of adult male WT (black; *n* = 12) and *Shank2-*KO (red; *n* = 10) mice (shading, SEM across mice) to strong (100 ms, 3 psi; left), mild (5 ms, 3 psi; middle), and very strong (100 ms, 30 psi; right) air puffs. Shaded rectangles indicate time periods before (green, 1.5 s before air puff onset), during (orange, 1 s after air puff onset), and after air puff delivery (purple, between 2.5 and 4 s after air puff onset). **B** The difference in eye closure response between *Shank2*-KO and WT mice to 16 different combinations of air puff duration (abscissa) and pressure (ordinate) before (left), during (middle), and after (right) air puff delivery. **C** Eye closure responses of adult of female WT (black; *n* = 11) and *Shank2*-KO (red; *n* = 10) mice. The results are presented as described for panels A–C. *p < 0.05, **p < 0.01, using two-way mixed ANOVA followed by post hoc Bonferroni test

Since ASD is associated with atypical sensory responses and heightened anxiety [93–95], we examined whether WT and *Shank2-*KO mice show differential eye closure responses to an air puff. Specifically, we delivered the air puff used in Task 1 (100 ms, 3 psi) without any preceding sensory cue (inter-trial interval, 9–11 s, uniform random distribution) and measured the fraction of eye closure before, during, and after air puff delivery (Fig. 2). Two-way mixed ANOVA revealed that there were significant main effects for genotype (*F*_*(1,40)*_ = 10.958, *p* = 0.004), time (*F*_*(2,40)*_ = 24.723, *p* = 1.0 × 10^–7^), and their interaction (*F*_*(2,40)*_ = 3.729, *p* = 0.033) on eye closure responses (Fig. 6A). Post hoc Bonferroni tests indicated that the eye closure response was significantly stronger in *Shank2*-KO than WT mice before (1.5-s time window before air puff onset; *p* = 0.001) and after (between 2.5 and 4 s since air puff onset; *p* = 0.005), but not during (1-s time window since air puff onset; *p* = 0.073) air puff delivery. These results indicate that anticipatory eye closure responses differ between adult male WT and *Shank2-*KO mice.

The above results raised the possibility that abnormal emotional responses to air puff may negatively affect reversal learning in male *Shank2-*KO mice. One way to explore this possibility would be to test male *Shank2-*KO mice using a mild air puff that does not induce an abnormal eye closure response. For this, we examined the relationship between air puff strength and eye closure response by systematically varying the duration and intensity of air puff (15 combinations other than the original one [100 ms, 3 psi]). Eye closure responses during air puff delivery (1-s time period since air puff onset) did not differ significantly between the two genotypes for any combination of air puff duration and intensity (*t* test, *p* values > 0.05; Fig. 6B). However, anticipatory eye closure responses before (1.5-s time window before air puff onset) and after (between 2.5 and 4 s since air puff onset) air puff delivery differed significantly between the two genotypes for some combinations of air puff duration and intensity (Fig. 6B). As expected, mild air puffs induced similarly low levels of anticipatory eye closure responses (Fig. 6A) in male WT and *Shank2-*KO mice. Based on these results, we chose to use the mildest air puff (5 ms, 3 psi; Fig. 6A and 6B) to further examine the reversal learning of male *Shank2-*KO mice.

Because female KO mice showed intact reversal learning in Task 1, we examined whether they would also show normal levels of eye close responses. In females, unlike males, *Shank2-*KO and WT mice showed similar levels of eye closure before, during, and after the delivery of the air puff used in Task 1 (100 ms, 3 psi; two-way mixed ANOVA, main effect of genotype, *F*_*(1,38)*_ = 0.057, *p* = 0.814; main effect of time, *F*_*(2,38)*_ = 109.621, *p* = 1.7 × 10^–16^; genotype × time interaction effect, *F*_*(2,38)*_ = 0.222, *p* = 0.802; Fig. 6C). We also failed to find a significant difference in eye closure response between female WT and *Shank2-*KO mice to mild (5 ms, 3 psi; main effect of genotype, *F*_*(1,38)*_ = 2.371, *p* = 0.140; main effect of time, *F*_*(2,38)*_ = 62.549, *p* = 9.5 × 10^–13^; genotype × time interaction effect, *F*_*(2,38)*_ = 0.279, *p* = 0.758; Fig. 6C) and very strong (100 ms, 30 psi; main effect of genotype, *F*_*(1,38)*_ = 0.002, *p* = 0.964; main effect of time, *F*_*(2,38)*_ = 47.561, *p* = 4.5 × 10^–11^; genotype × time interaction effect, *F*_*(2,38)*_ = 0.274, *p* = 0.762; Fig. 6C) air puffs. These results indicate that female *Shank2-*KO mice lack the heightened fear response observed in male *Shank2-*KO mice.

### Intact reversal learning of male Shank2-KO mice in the presence of mild air puff

**Fig. 7:**
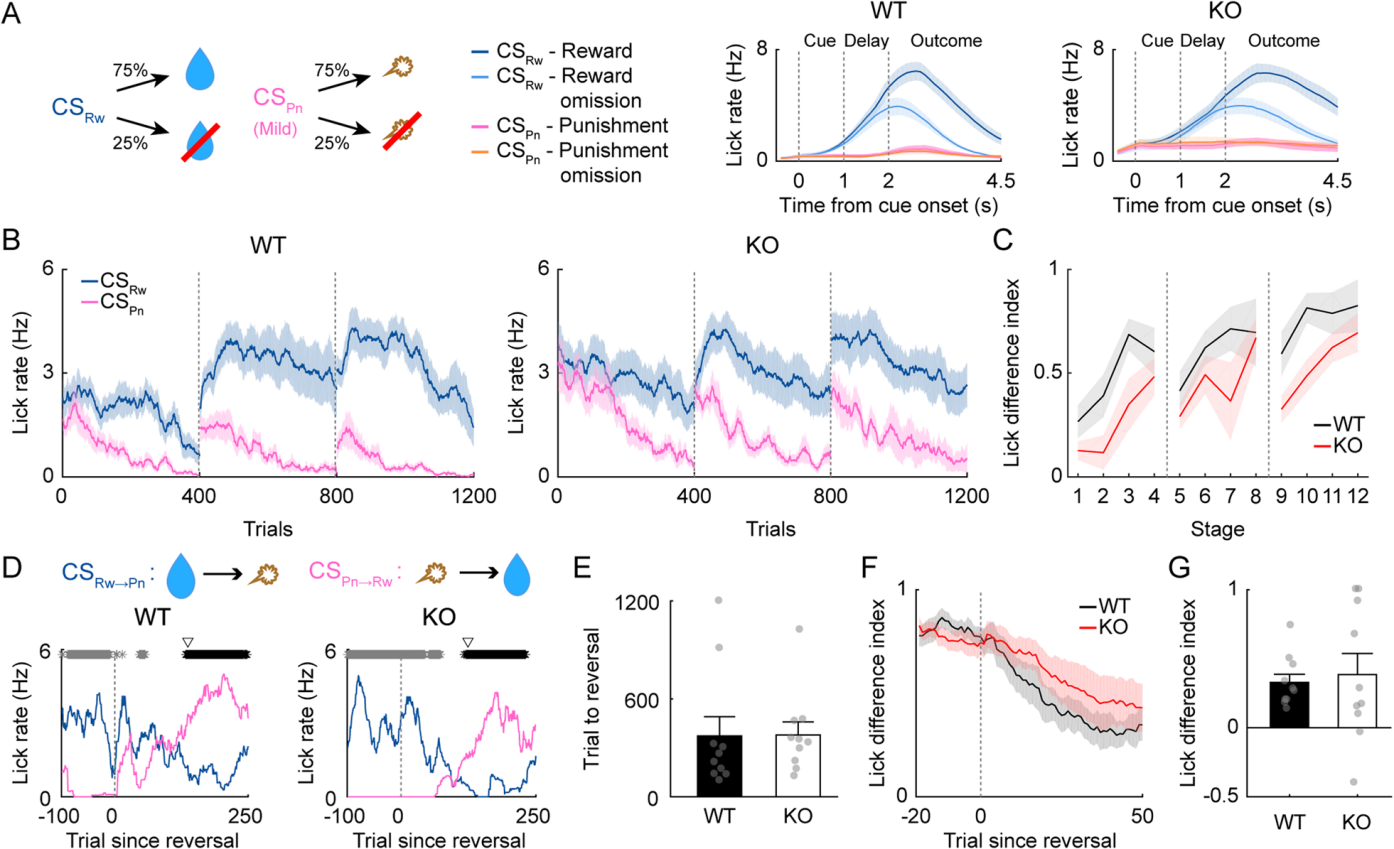
Adult *Shank2-*KO mice exhibit intact reversal learning with the use of a mild air puff. Results of reversal training with a mild air puff (Task 2) in adult male WT (*n* = 10) and *Shank2*-KO (*n* = 10) mice. The results are presented as described for Fig. 3A–G, except that pink lines indicate anticipatory lick rates in response to the CS_Pn_ predicting a mild air puff (5 ms, 3 psi)

Using the mildest air puff (5 ms, 3 psi), to which adult male WT and *Shank2-*KO mice showed similar anticipatory eye closure responses, we tested another group of adult male mice (10 WT and 10 *Shank2-*KO) for reversal learning (Task 2; Fig. 7A). Both male WT and *Shank2-*KO mice quickly developed and maintained preferential anticipatory licking responses to CS_Rw_ versus CS_Pn_ during the initial training sessions. Two-way mixed ANOVA of LDI revealed that there was a significant main effect of training (*F*_*(11,187)*_ = 14.010; *p* = 1.9 × 10^–19^) and genotype (*F*_*(1,187)*_ = 14.561; *p* = 0.001), but no significant interaction effect between the two (*F*_*(11,187)*_ = 1.092; *p* = 0.370; Fig. 7B, C). Thus, both genotypes learned the CS-US contingencies well, but the initial learning was stronger in WT mice than in *Shank2-*KO mice.

The mice were then trained until they reached the reversal criterion over 2–4 daily sessions (400 trials each). The number of trials needed to reach the reversal criterion did not vary significantly between the male WT and *Shank2-*KO mice (373.6 ± 117.5 and 379.0 ± 79.9 trials, respectively; *t* test, *t*_*(18)*_ = 0.038, *p* = 0.970; Fig. 7D, E; similar results were obtained after deletion of trials in which previously delivered water was consumed; 114 out of 7697 trials; 1.48%; *t* test, *t*_*(18)*_ = 0.084, *p* = 0.934). The LDI (calculated using trials #41–50 of the first reversal session) also did not differ significantly between the two genotypes (0.328 ± 0.059 and 0.386 ± 0.153, respectively; *t* test, *t*_*(18)*_ = 0.348, *p* = 0.732; Fig. 7F, G). These measures of reversal learning had no significant relationship with the number of licks in CS_Rw_ trials, the number of licks in CS_Pn_ trials, the difference in the number of licks between CS_Rw_ and CS_Pn_ trials, or the LDI during the last session of the initial training (Additional file 1: Fig. S3). These results indicate that male *Shank2-*KO mice exhibit intact reversal learning in Task 2. Similar results were obtained with juvenile (P30–45) male WT and *Shank2-*KO mice tested on Task 2 (Additional file 1: Fig. S4).

### Intact reversal learning of Shank2-KO mice in the absence of aversive outcome

**Fig. 8:**
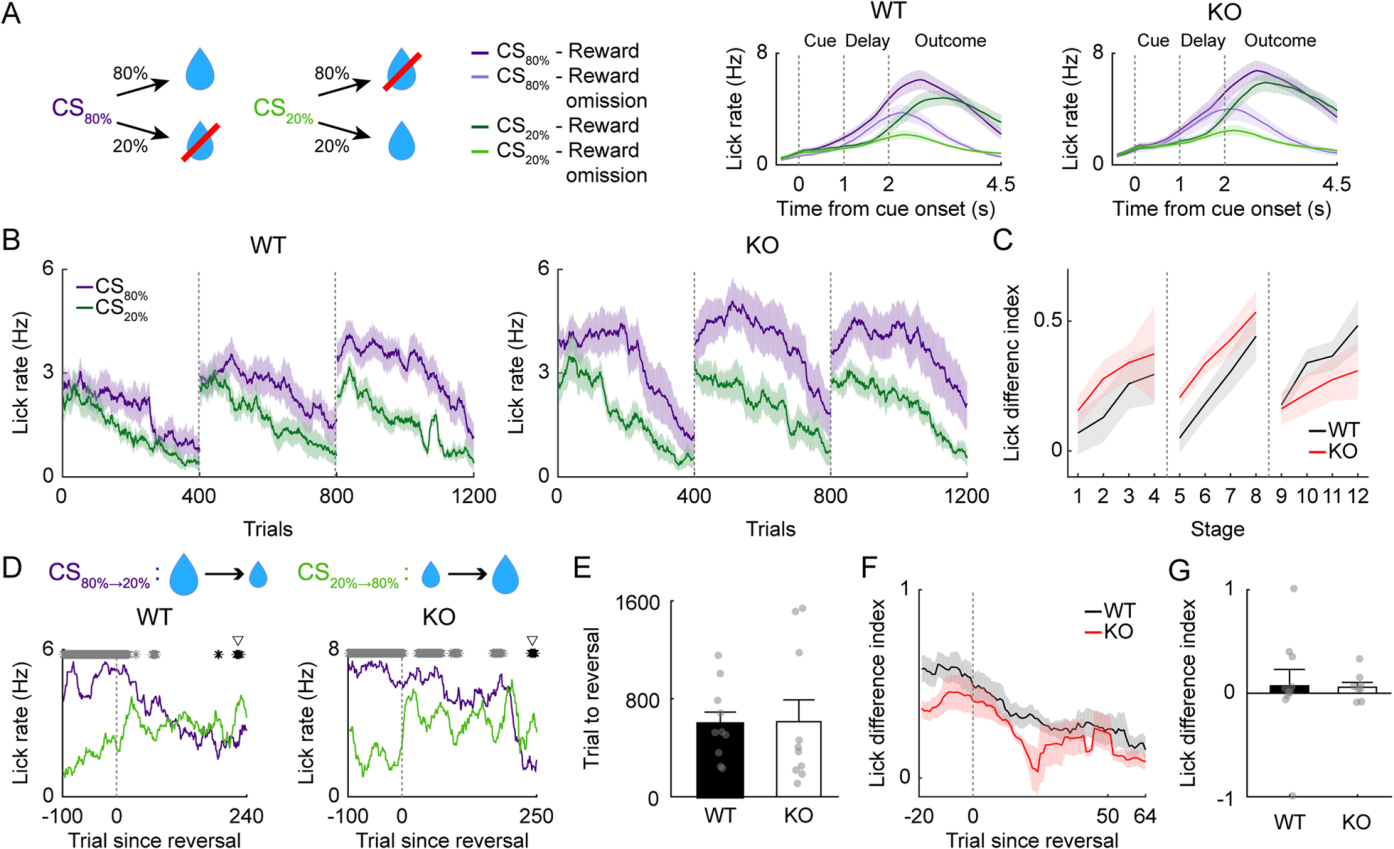
*Shank2-*KO mice exhibit intact learning in reward probability reversal. Results of reversal training using only appetitive outcomes (Task 3) in adult male WT (*n* = 10) and *Shank2*-KO (*n* = 10) mice. The results are presented as described for Fig. 3A–G, except that the purple and green lines denote anticipatory lick rates in response to CS_80%_ and CS_20%_, respectively

To further confirm that the behavioral flexibility of male *Shank2-*KO mice is intact in the absence of a strong aversive outcome, we tested another group of adult male WT (*n* = 10) and *Shank2-*KO (*n* = 10) mice in reversal learning using only appetitive outcomes. In Task 3, two different odor cues were paired with the same amount of water (6 µl), but with two different probabilities (80 and 20%; CS_80%_ and CS_20%_, respectively; Fig. 8A). In both genotypes, anticipatory licking responses were higher to CS_80%_ than CS_20%_ throughout the acquisition session (Fig. 8B). Two-way mixed ANOVA of LDI revealed that there was a significant main effect of training (*F*_*(11,198)*_ = 5.117; *p* = 4.8 × 10^–7^) but no significant main effect of genotype (*F*_*(1,198)*_ = 0.447; *p* = 0.513) or training × genotype interaction effect (*F*_*(11,198)*_ = 1.329; *p* = 0.211; Fig. 8C).

During the reversal training (2–4 daily sessions of 400 trials until the reversal criterion), the number of trials needed to reach the reversal threshold did not differ significantly between the male WT and *Shank2-*KO mice (594.4 ± 97.1 and 614.2 ± 177.4 trials, respectively; *t* test, *t*_*(18)*_ = 0.098, *p* = 0.923; Fig. 8E; similar results were obtained after deletion of trials in which previously delivered water was consumed; 210 out of 10,686 trials; 1.97%; *t* test, *t*_*(18)*_ = 0.950, *p* = 0.355). Also, the LDI (calculated using trials #53–64 of the first reversal session) did not differ significantly between the male WT and *Shank2-*KO mice (0.207 ± 0.061 and 0.108 ± 0.042, respectively; *t* test, *t*_*(16)*_ = 0.076, *p* = 0.940; Fig. 8F, G), and these reversal learning measures had no significant relationship with the number of licks in CS_80%_ trials, the number of licks in CS_20%_ trials, the difference in the number of licks between CS_80%_ and CS_20%_ trials, or the LDI during the last acquisition session (Additional file 1: Figure S5). These results verify that the reversal learning of *Shank2-*KO mice is intact in the absence of a strong aversive outcome.

### Effect of DCS on fear response

**Fig. 9:**
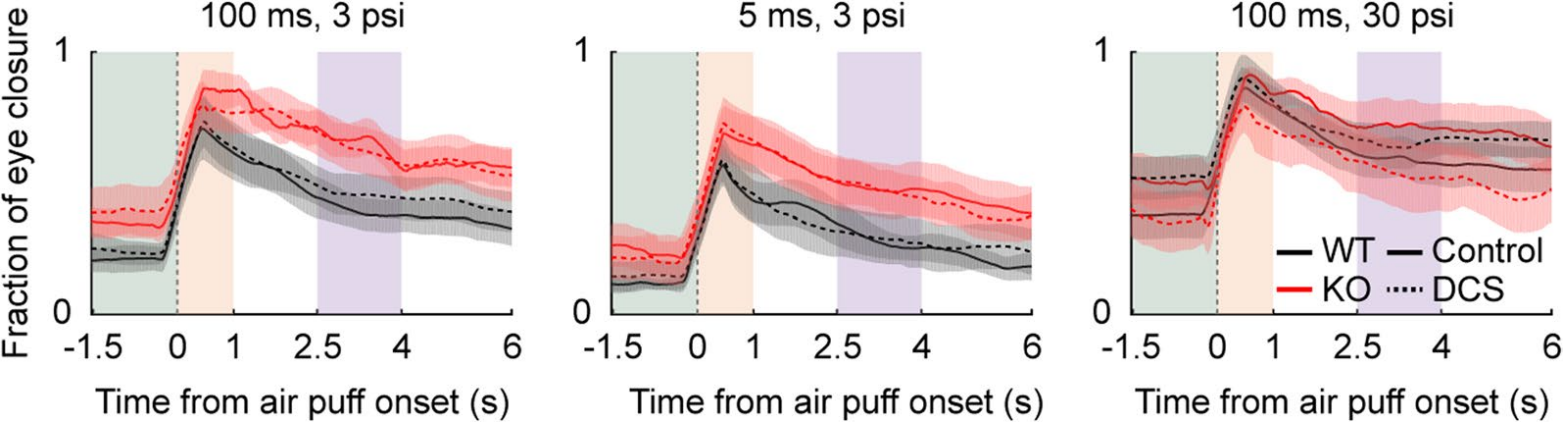
DCS does not rescue the abnormal eye closure response of male *Shank2-*KO mice. Eye closure responses (average of 5 trials) of adult male WT (black; *n* = 11) and *Shank2-*KO (red; *n* = 10) mice (shading, SEM across mice) treated with DCS (dashed line) or saline (solid line) injection and exposed to the strong, mild, and very strong air puffs

Previous studies [60, 67] showed that DCS, a partial agonist of NMDA receptor, rescues the social interaction deficits of *Shank2*-KO mice. We therefore tested whether DCS would also rescue the behavioral deficits of *Shank2*-KO mice found in the current study. As repeated administration of DCS causes tachyphylaxis [96–99] and the drug’s half-life in mice is only 23 min [100, 101], it would have been difficult to test the effects of DCS on the above-described reversal learning, which takes a relatively long time (1–5 days of training). Given that our results suggested that enhanced fear is the source of reversal learning deficit in male *Shank2*-KO mice, and eye close responses can be tested within a short period of time, we examined whether DCS could rescue the abnormal eye closure responses (i.e., fear responses) of adult male *Shank2*-KO mice. We tested the effects of DCS on eye closure responses to the strong (100 ms, 3 psi), mild (5 ms, 3 psi), and very strong (100 ms, 30 psi) air puffs. For the strong air puff (100 ms, 3 psi), three-way mixed ANOVA revealed that there were significant main effects of genotype (*F*_*(1,38)*_ = 4.714, *p* = 0.043) and time (*F*_*(2,38)*_ = 45.503, *p* = 8.2 × 10^–11^), but no significant interaction between them (*F*_*(2,38)*_ = 0.719, *p* = 0.494). The main effect of drug (*F*_*(1,38)*_ = 0.122, *p* = 0.731) and the other interaction effects were not statistically significant (genotype × time, *F*_*(2,38)*_ = 0.719, *p* = 0.494; drug × genotype, *F*_*(1,38)*_ = 0.130, *p* = 0.723; drug × time, *F*_*(2,38)*_ = 0.331, *p* = 0.720; drug × genotype × time, *F*_*(2,38)*_ = 0.489, *p* = 0.617; Fig. 9A). Similarly, the main effect of drug and the effects of interactions involving the drug were not statistically significant for the mild (main effect of drug, *F*_*(1,38)*_ = 0.020, *p* = 0.888; drug × genotype, *F*_*(1,38)*_ = 0.012, *p* = 0.914; drug × time, *F*_*(2,38)*_ = 0.050, *p* = 0.951; drug × genotype × time, *F*_*(2,38)*_ = 0.189, *p* = 0.829; Fig. 9B) and very strong (main effect of drug, *F*_*(1,38)*_ = 0.186, *p* = 0.671; drug × genotype, *F*_*(1,38)*_ = 2.888, *p* = 0.106; drug × time, *F*_*(2,38)*_ = 0.362, *p* = 0.700; drug × genotype × time, *F*_*(2,38)*_ = 0.559, *p* = 0.577; Fig. 9C) air puffs. These results indicate that DCS does not rescue the enhanced fear response of male *Shank2*-KO mice to the air puff.

## Discussion

We herein examined the behavioral flexibility of *Shank2-*KO mice by testing their capability for reversal learning in a probabilistic trace classical conditioning paradigm using the anticipatory lick rate as an index for learning. Compared to WT mice, male *Shank2-*KO mice showed significantly slower reversal learning when a water reward and a strong air puff were used as the appetitive and aversive outcomes, respectively (Task 1). Moreover, male *Shank2-*KO mice showed stronger eye closure responses than WT mice to the anticipated air puff. On the one hand, eye closure responses did not vary significantly between male *Shank2-*KO and WT mice immediately after an air puff was delivered. This suggests that the reversal learning deficit of *Shank2-*KO mice observed in Task 1 is unlikely to reflect abnormal sensory processing, which is often observed in people with ASD [102, 103] and animal models of ASD [103, 104], or abnormal reactivity to an aversive stimulus. On the other hand, male *Shank2-*KO mice showed significantly stronger eye closure responses than WT mice before and after air puff delivery, indicating that repeated exposure to strong air puffs altered the basal level of eye closure in these mice. Both nonspecific emotional (fear) and specific motor (eyelid movement) responses develop during eyeblink conditioning. Conditioned fear, such as an increase in blood pressure/heart rate and pupillary dilation, emerges only after a few CS-US pairings, whereas conditioned eyeblink (with the peak responses precisely timed to the expected delivery of the US) typically emerge after many CS-US pairings [105–107]. Our results are consistent with the idea that male *Shank2-*KO mice show enhanced fear to an anticipated aversive stimulus, rather than an enhanced reflexive motor response to the delivery of an aversive stimulus. This elevated fear may suppress the behavioral expression of learned cue-outcome contingencies during reversal training in Task 1.

Consistent with this possibility, when we replaced the strong air puff with a mild air puff that induced similar anticipatory eye closure responses between male *Shank2-*KO and WT mice, male *Shank2-*KO mice showed intact reversal learning (Task 2). Male *Shank2-*KO mice also showed intact reversal learning between two cues predicting rewards with different probabilities (Task 3). Together, these results are consistent with the notion that male *Shank2-*KO mice show enhanced fear but intact learning of cue-outcome contingency changes in our behavioral settings. This is further supported by the finding that female *Shank2-*KO mice were intact in reversal learning in the presence of a strong air puff and also showed WT-level anticipatory eye closure responses to the strong air puff. A previous study found that reversal learning was delayed in people with ASD in a fear conditioning paradigm in which an air puff was used as an US [15]. Our results raise the possibility that people with ASD might show intact reversal learning if the US is replaced with a mild air puff, which remains to be tested. Our present results suggest that the neural machinery needed to keep track of changes in cue-outcome contingency and to control behavior according to such evaluations are likely to be intact in these brain structures of *Shank2-*KO mice, but their behavioral expressions are suppressed by enhanced fear in male *Shank2-*KO mice.

Previous studies on reversal learning in ASD model mice yielded mixed results. Both intact and impaired reversal learning have been reported for a diverse array of ASD mouse models in diverse tasks. Regarding classical conditioning using aversive outcomes, *Tsc1* mutant mice were reported to be impaired in the reversal of eyeblink conditioning [24]. Given that *Tsc1* and *Tsc2* form a complex for signal transduction and *Tcs2* deletion increases anxiety [108], the impairment of Tsc1 mutant mice in the reversal of eyeblink conditioning is in line with our finding that an altered emotional response may limit behavioral flexibility in a mouse model of ASD. Some previous studies found that reversal learning is intact in ASD model mice exposed to tasks involving no aversive outcome. For example, mice with mutations in postsynaptic density protein-95 were intact in reversal learning on a touch screen task in which one target was associated with a reward and another with no reward [109]. These results are consistent with our finding that *Shank2*-KO mice were intact in reversal learning when only appetitive outcomes were used. However, numerous other studies found that reversal learning was impaired in various ASD model mice in the absence of an aversive outcome [16–20, 22–29]. It is difficult to compare findings across studies because the studied genotypes and experimental procedures have varied widely. For example, the utilized experimental procedures have varied in their choice availability (classical vs. instrumental tasks), outcome valence (appetitive vs. aversive), reinforcement type (positive vs. negative), outcome certainty (deterministic vs. probabilistic delivery), freedom of movement (head-fixed vs. freely moving), stimulus modality (olfactory, visual, etc.), and choice modality (spatial vs. nonspatial). These factors may directly or indirectly influence performance in reversal learning. For example, anxiety is elevated under uncertainty [110, 111] and anxiety disorder patients prefer to play a passive rather than an active role in decision-making [112]. Thus, it is possible that uncertain outcomes under a free-choice condition may elevate anxiety and thereby impair reversal learning in certain ASD model mice. Consistent with this, BTBR T + Itpr3tf/J and C58 mice showed intact reversal learning when a reward was delivered in an all-or-none manner, but exhibited impaired reversal learning when the reward was delivered probabilistically [17, 23]. Our results raise the possibility that altered emotional responses may limit behavioral flexibility despite the presence of intact learning capability in ASD.

*Shank2*-KO mice lacking exons 6 and 7, such as those used in the present study, show reduced NMDA receptor (NMDAR) function in several brain regions, including the medial prefrontal cortex, hippocampus, and amygdala, at juvenile and adult stages [60, 67, 68, 71]. This NMDAR hypofunction has been causally associated with paradoxically increased NMDAR function at early postnatal stages [67], which highlights the long-lasting impacts of early postnatal pathophysiology [83]. Normalizing the NMDAR hypofunction in *Shank2*-KO mice by direct DCS-dependent NMDAR activation or by indirect NMDAR activation (through the zinc chelator, clioquinol, or early postnatal memantine treatment) rescues mainly social deficits but not other behavioral deficits, including hyperactivity, repetitive behaviors, and anxiety-related behaviors [60, 67, 71, 113]. Whether fear responses are altered by and associated with NMDAR dysfunction has not been tested in our *Shank2*-KO mice or other *Shank2*-KO mouse lines [65]. Nevertheless, our present finding that DCS does not alter the eye closure fear responses of *Shank2*-KO mice does not seem to disagree with the previous report that NMDAR activation has the main effect on social rescue. Currently, Shank2-related circuit dysfunctions remain largely unexplored [68], partly because Shank2 is widely expressed in various brain regions, including the cerebellum [32, 61, 62], and in various cell types, including excitatory and inhibitory neurons [68]. Clearly, further studies are needed to elucidate the critical Shank2-related circuit dysfunction that leads to enhanced fear.

The male–female difference in the reversal learning and anticipatory eye closure responses in *Shank2*-KO mice is intriguing in light of previous reports that male and female *Shank2*-KO mice display similar NMDAR hyperfunction and behavioral deficits in social, repetitive behavioral, locomotor, and anxiety-like domains as well as similar pharmacological rescue profiles of these deficits [60, 67]. However, male–female differences in mouse models of ASD have been differentially detected in various behavioral, synaptic, molecular, and neuroanatomical phenotypes [77–82]. Also, male–female differences in eyeblink conditioning have been reported in rats, mice, and humans [114–116]. Therefore, it is possible that reversal learning and fear responses represent two behavioral tests through which male–female differences in *Shank2*-KO mice may be detected. Although the related mechanisms remain to be determined, previous transcriptomic studies on human and mouse samples have identified sexually dimorphic expression patterns among astrocyte- and microglia-related genes, suggesting that there are sex differential interplays between neuronal and glial cells [76, 77, 117].

## Limitations

Our study has limitations in many respects. First, our conclusions are drawn from the results obtained from only one mouse model of ASD (*Shank2*-KO mice) tested in one type of behavioral task (reversal learning in a probabilistic trace classical conditioning paradigm). Therefore, future work is needed to clarify the extent to which altered emotional responses contribute the behavioral inflexibility associated with ASD. Second, it remains possible that abnormalities in processes other than emotional processing contribute to the behavioral inflexibility seen in ASD. We examined only the influence of enhanced fear on reversal learning; meanwhile, behavioral flexibility can be caused by abnormalities in many different underlying processes, such as inhibitory control, value-based decision-making under free-choice conditions, and the ability to capture complex stimulus–response–outcome contingencies (task rules) under cognitively demanding situations. In this regard, our results do not argue directly against the cognitive inflexibility hypothesis for ASD [13]. Third, we did not investigate the neurobiological mechanisms linking *Shank2-*KO and enhanced fear in this study. The brain structures involved in the fear-induced suppression of reversal learning in male *Shank2-*KO mice are unclear, as are the physiological and molecular processes leading to enhanced fear in male *Shank2-*KO mice. All of these details remain to be elucidated.

## Conclusions

Our results demonstrate that one consequence of *Shank2*-KO in male mice is an abnormally heightened fear that may limit behavioral flexibility under certain circumstances. Our findings suggest that behavioral flexibility may be seriously limited by abnormal emotional responses in ASD. Further studies are needed to determine the extent to which people with ASD and animal models of ASD show impairments in their ability to flexibly adjust behavior due to altered emotional responses under diverse behavioral settings. Going forward, the neurobiological mechanisms linking *Shank2*-KO and enhanced fear remain to be determined.

## Supplementary Information

**Additional file 1: Table S1, Table S2, Fig. S1, Fig. S2, Fig. S3, Fig. S4 and Fig. S5. **Statistical test results for eyelid closure responses to diverse combinations of air puff duration and pressure.

## Data Availability

Raw data and code to reproduce this work are archived at Mendeley Data (https://data.mendeley.com/datasets/bg8s4zpdk6/1).

## Abbreviations

ASD: Autism spectrum disorder
CS: Conditioned stimulus
DCS: D-cycloserine
KO: Knockout
LDI: Lick difference index
PCR: Polymerase chain reaction
Shank2: SH3 and multiple ankyrin repeat domains 2
US: Unconditioned stimulus
WT: Wild-type
