# Supplementary material for: Enhanced fear limits behavioral flexibility in Shank2-deficient mice
Source: Mol Autism. 2022 Oct 3;13:40. doi: 10.1186/s13229-022-00518-1 (PMC9531513; doi:10.1186/s13229-022-00518-1)
Supplement: Supplementary file 1 — Additional file 1: Table S1, Table S2, Fig. S1, Fig. S2, Fig. S3, Fig. S4 and Fig. S5. Statistical test results for eyelid closure responses to diverse combinations of air puff duration and pressure. [file 13229_2022_518_MOESM1_ESM.pdf]

Enhanced fear limits behavioral flexibility in *Shank2*-deficient mice

Supplement

Table S1. The numbers of subjects used in each experiment. For those mice tested in reversal learning (Tasks 1–3), the number in each parenthesis indicates the number of mice tested in eye close responses before being test in reversal learning. For those mice tested in eye closure response, the number in each parenthesis indicates the number of mice that were later tested in reversal learning.

	**Adult male WT**	**Adult male KO**	**Adult female WT**	**Adult female KO**	**Juvenile male WT**	**Juvenile Male KO**
**Task 1**	10	10(1)	12(11)	10(3)	10	11
**Task 2**	10	10			10	11
**Task 3**	10(8)	10(4)				
**Eye response**	12(8)	10(5)	11(11)	10(3)		
**Rescue**	11	10				
**Total**	45	45	12	17	20	22

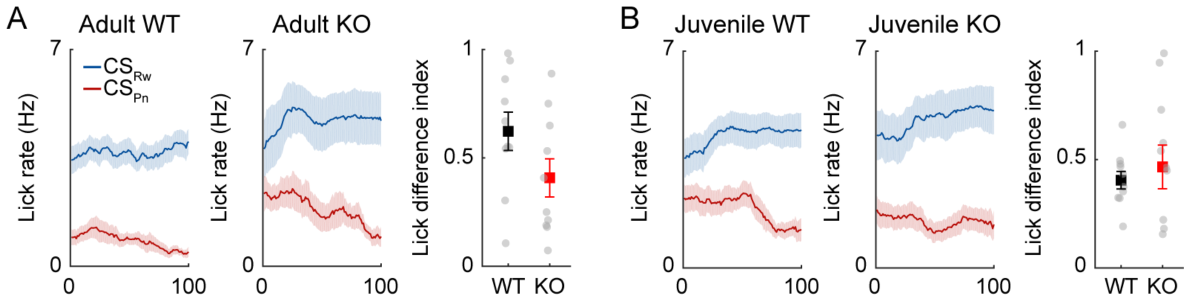

Supplementary figure 1. Anticipatory licking behavior is similar between adult and juvenile male mice before reversal onset. Shown are anticipatory lick rate profiles (mean±SEM across mice) of adult and juvenile male WT and *Shank2*-KO mice during the first 100 trials before reversal onset (left and middle) and the 100-trial averages (right; circles, individual animal data; square and error bar, mean±SEM across mice) in the first reversal session. Two-way ANOVA of anticipatory lick rates revealed no significant age or genotype effect (main effect of age, $F_{(1,37)}$ = 0.907, p = 0.347; main effect of genotype, $F_{(1,37)}$ = 0.835, p = 0.367; age×genotype interaction, $F_{(1,37)}$ = 2.707; p = 0.108). Two-way ANOVA of the number of trials needed to reach the reversal criterion revealed no significant age or genotype effect, either (main effect of age, $F_{(1,37)}$ = 1.811, p = 0.187; main effect of genotype, $F_{(1,37)}$ = 1.572, p = 0.218; age×genotype interaction, $F_{(1,37)}$ = 0.070; p = 0.793). These results indicate that juvenile WT and *Shank2*-KO mice, like adult mice, were well trained in the task before reversal onset.

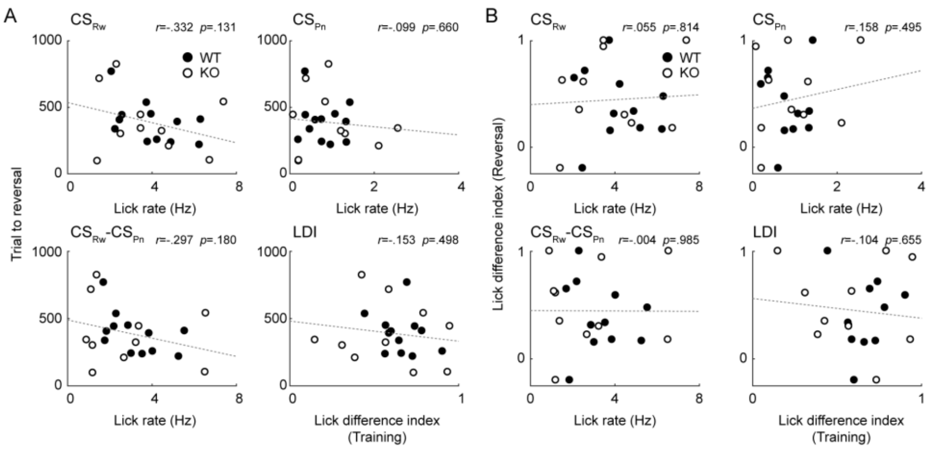

Supplementary figure 2. No significant relationship between reversal performance and acquisition performance in female mice. Results of adult female WT (n = 12) and *Shank2*-KO (n = 10) mice in Task 1 reversal learning. The results are presented as described for Fig. 3H, I.

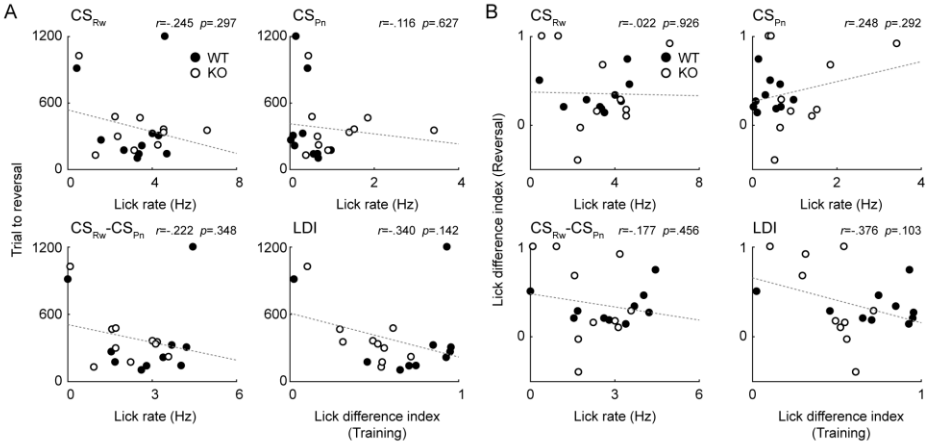

Supplementary figure 3. No significant relationship between reversal performance and acquisition performance in Task 2 reversal learning. Results of adult male WT (n = 10) and *Shank2*-KO (n = 10) mice. The results are presented as described for Fig. 3H, I.

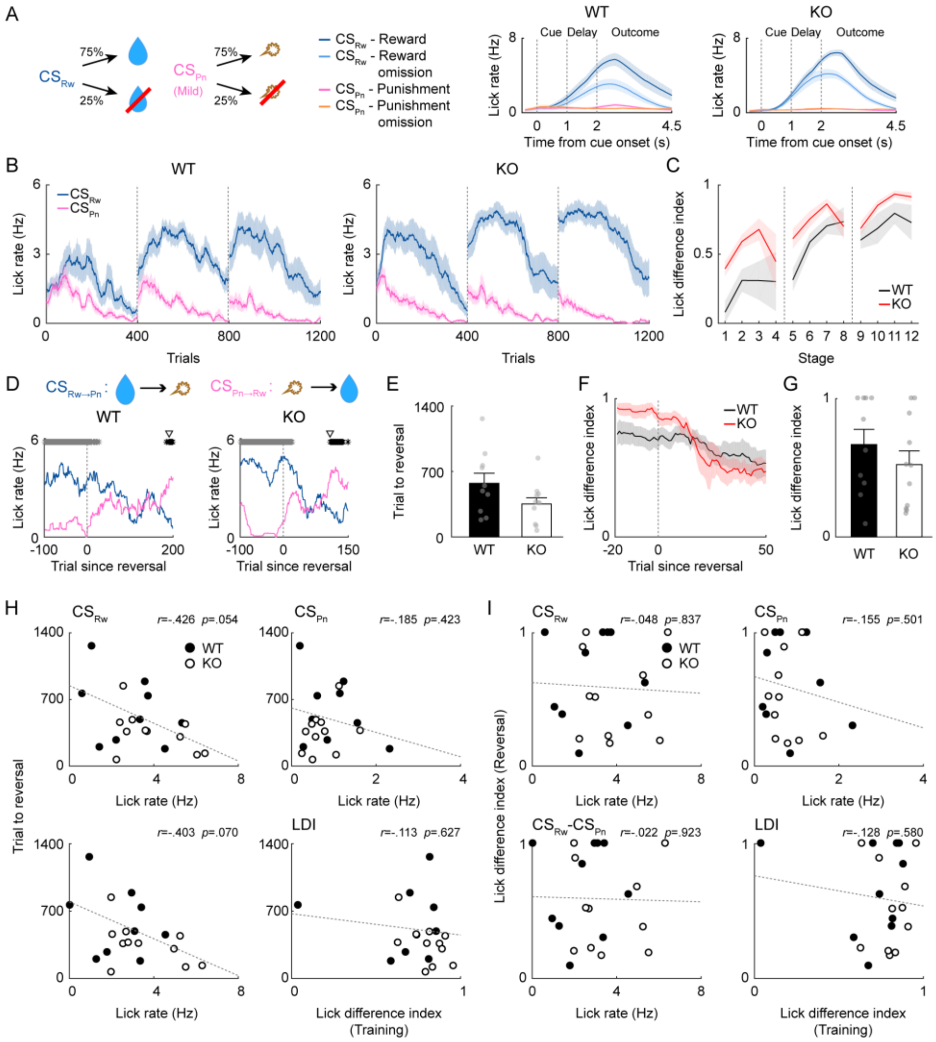

Supplementary figure 4. Juvenile *Shank2*-KO mice exhibit intact learning in reward-probability reversal. Results of reversal training with a mild air puff (5 ms, 3 psi; Task 2) in juvenile male WT (n = 10) and *Shank2*-KO (n = 11) mice in Task 2. The results are presented as described for Fig. 3.

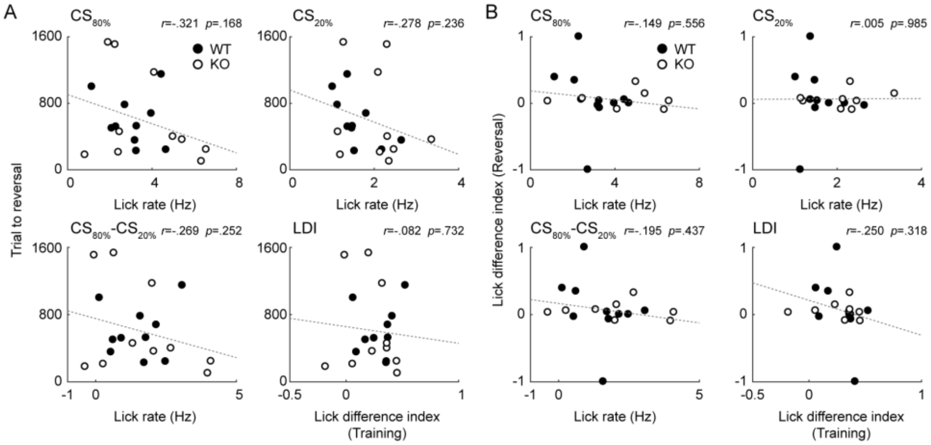

Supplementary figure 5. No significant relationship between reversal performance and acquisition performance in reward-probability reversal. Results of adult male WT (n = 10) and *Shank2*-KO (n = 10) mice in Task 3. The results are presented as described for Fig. 3H, I.
